# Use of Sub-Atmospheric Pressure Storage to Improve the Quality and Shelf-Life of Marmande Tomatoes cv. Rojito

**DOI:** 10.3390/foods12061197

**Published:** 2023-03-12

**Authors:** María del Carmen Salas-Sanjuán, María del Mar Rebolloso, Fernando del Moral, Juan Luis Valenzuela

**Affiliations:** 1 Department of Agronomy, Higher Engineering School, Research Centres CIAIMBITAL and CeiA3, University of Almería, 04120 Almería, Spain; 2 Department of Biology & Geology, Higher Engineering School, Research Centres CIAIMBITAL and CeiA3, University of Almería, 04120 Almería, Spain

**Keywords:** fruit quality, postharvest life, *Solanum lycopersicum*, sub-atmospheric storage

## Abstract

In this study, the feasibility of storing Marmande tomatoes (*Solanum lycopersicum,* cv Rojito) under hypobaric conditions was evaluated. The fruits were sorted into four lots of 72 fruits each. One lot was considered as a control, and the fruits were kept in the open box, while the fruits of the rest of the three remaining lots were enclosed in airtight containers and subjected to 101, 75 and 50 Kpa, respectively. Control fruits and airtight containers were kept at room temperature, and every three days from the beginning of the experiment the following main quality parameters were analysed: ethylene production rate, firmness, colour, total solids content, ascorbic acid, total phenolics and pigments, as well as a sensory analysis carried out by panellists. The results show that sub-atmospheric storage led a reduction in ethylene production, which was associated with a delay in ripening. The differences in the evolution of pigments were very significant, while a large degradation of chlorophylls was observed in the control fruits and in those kept at 101 kPa, in the fruits kept at 75 kPa and 50 kPa the degradation was much slower. In relation to carotenoid pigments, it was observed that sub-atmospheric treatments delayed their appearance compared to control and 101 kPa fruits. In relation to other quality parameters, it was found that control fruit and fruit held at 101 kPa softened more rapidly than fruit under sub-atmospheric conditions, whose loss of firmness was more gradual with differences found only at 9 and 12 days of storage with respect to fruit firmness at harvest. The appearance of these fruits was evaluated with the same score as at the time of harvesting, during 9 of the 12 days of the experiment, then a positive effect of sub-atmospheric treatments was also found in the sensory analysis. The results suggest that sub-atmospheric storage could be a suitable method of increasing the shelf-life of fruits.

## 1. Introduction

Tomato (*Solanum lycopersicum* Mill.) is one of the most widespread and important vegetable crops in all regions of the world, and its worldwide production exceeds 130 million tonnes. Tomatoes must have taste, colour, texture, and organoleptic qualities capable of satisfying consumers. In addition to these requirements, they must also be suitable for postharvest handling, transport, and marketing [1]. The different cultivars of the Marmande type, such as RAF, Rojito, Adora and Delizia, are traditional crops in the province of Almería characterised by high organoleptic quality, high market price and very short postharvest life as a result of the rapid development of all the ripening processes after harvesting [2,3,4]. This short postharvest life is a hinderance to the marketing of these fruits, which are usually consumed in the breaker or turning ripening stage. It is at these stages of ripening that the fruits show all their quality attributes, such as firm flesh, high sugar content and a balanced flavour.

For these reasons, different techniques have been used to increase the shelf life of Marmande tomatoes, maintaining their firmness, as well as the rest of the quality attributes. For example, ethylene antagonists, such as 1-MCP [4], oil-based postharvest regulators [2] and modified atmospheres [5] have been used for this purpose. Although there are various techniques used for the preservation of horticultural products, the storage under hypobaric conditions is very little applied. This technique was introduced by Burg and Burg in 1966 [6] and consists of storing horticultural products under sub-atmospheric pressure conditions, generally 50 kPa. As a consequence of respiration, the oxygen concentration may drop to levels where anaerobic conditions occur, so the containers are regularly opened and flushed with fresh air, and then hypobaric conditions are re-established. Since then, various assays have been carried out to improve the quality and preservation of asparagus [7], lettuce and strawberry [8], pears [9], tomatoes [10,11,12], and other horticultural products [13,14] and some tropical fruits as mango [15,16].

The application of this technique has positive effects on the delay of senescence in different fruits and vegetables. The effects are centred on the maintaining firmness and colour as well as a delay in the loss of organoleptic quality. Moreover, a delay in the degradation of chlorophyll contributes to an increase in postharvest life. In mango, ripening was delayed when the fruit was maintained at 13 kPa, prolonging shelf life [16]. It was found that shelf-life extension was inversely proportional to pressure, although pressures below 7 kPa caused dehydration, leading to fruit shrivelling [17]. Under hypobaric conditions, the partial pressure of oxygen is reduced, and this leads to a slower rate of aerobic respiration, which reduces the catabolism of respiratory substrates and the generation of energy required for the biochemical reactions associated with fruit ripening. The same effect has been found in strawberry and cut lettuce leaves, where the rate of respiration markedly decreased when kept under sub-atmospheric conditions [8]. Hypobaric treatments have also been used to inhibit and/or reduce losses and spoilage due to fungal infections in grapes, cherries, and strawberries, indicating that it could induce resistance against pathogens [17]. Fruit storage under an atmosphere with a low partial pressure of oxygen slows down the metabolism of fruit, thus lowering the production of ethylene. In addition to this lower ethylene production, the renewal of the air allows the elimination of the ethylene produced, resulting in a delay in ripening processes and a longer postharvest life. During the ripening of the fruits, metabolic reactions occur that are characterised by the production of reactive oxygen species (ROS). Nevertheless, ROS production depends on the balance between the production of ROS and ROS-scavenging systems [18,19,20]. A positive effect of hypobaric storage has also been found on the antioxidant system that protects fruits from (ROS). As a result, the accumulation of ROS decreases, and thus senescence is delayed [21,22].

However, hypobaric storage technology has not been widely used in the industry, possibly due to investment costs and a lack of knowledge about the benefits of hypobaric storage technology. This has led to few companies offering hypobaric storage services [23,24]. Portable vacuum packing machines are widely used at home and in food retail outlets. These machines often use embossed vacuum bags, but rigid airtight containers are also used to maintain sub-atmospheric pressures. The storage of fresh fruit and vegetables in these domestic devices is similar to conventional hypobaric storage, although there are marked differences. The main difference is that the sub-atmospheric pressures reached in domestic devices are higher than in industrial hypobaric storage. Thus, while pressures of 70 or 50 kPa are normal in a domestic vacuum sealer, in industrial vacuum storage equipment, the pressures reached are usually lower than 10 kPa. In addition, domestic equipment has no automatic air freshening system.

Little research has been conducted on the use of sub-atmospheric packaging for household devices, but Ann et al. [8] found that this type of packaging reduces the respiration rate in lettuce. This suggests that sub-atmospheric packaging could be beneficial for preserving the freshness of fruits and vegetables; however, due to the large surface to volume ratio in leafy vegetables, as well as the presence of stomata, high water loss is found to limit the effectiveness of storage. On the contrary, for fruits such as strawberries, tomatoes and others, the results are satisfactory [8,12,24].

This study details the efficacy of the sub-atmospheric storage of Marmande tomato, cultivar Rojito, as a method of increasing postharvest life and maintaining fruit quality. In addition, the evolution of the main physiological and fruit quality parameters was evaluated, as well as the composition of bioactive compounds in the fruit.

## 2. Materials and Methods

### 2.1. Materials and Experimental Design

Fresh market tomatoes (*Solanum lycopersicum* L. cv Rojito) were supplied by Biosabor, S.A.T. and grown in a greenhouse according to the cultural practices typical of this crop. The fruits were harvested by hand at the turning maturity stage in accordance with the USDA standard tomato colour chart [25]. The fruits were sorted into four lots of 72 fruits each, all fruits being uniform in size and ripening stage and free of damage and deformities. One lot was considered a control group, where the fruits were not packaged and were kept in the open box. The fruits of a second batch were stored in 24 airtight containers of two litters each, with each container having a similar weight–volume ratio of 425 ± 25 g/2L (three fruits per container), and then the containers were closed and kept at atmospheric pressure (101 kPa) considered (treatment T-101 kPa). The fruits of the third lot were distributed as in the previous lot but subjected to a sub-atmospheric pressure of 75 kPa (treatment T-75 kPa). Finally, the fruits of the fourth batch were subjected to a pressure of 50 kPa (treatment T-50 kPa). All the fruits were maintained at room temperature (20 ± 2 °C), and to avoid anoxia conditions every 24 h, the air was renewed in all the containers and then closed, maintaining the respective pressure of each lot according to the methodology devised by An et al. [8]. The airtight containers are made of rigid plastic material and had a valve to which a semi-professional vacuum packaging machine with a manometer and a pump flow rate of 15 L/min (Garhe, vacuum system model 31370, Amorebieta, Vizcaya, Spain) was connected, helping to maintain sub-atmospheric pressures inside the containers. Every three days from the beginning of the experiment, six containers per treatment (18 fruits) were used to perform the analyses. In the same way, 18 fruits were randomly selected from the control treatment. The initial quality of the fruits was determined on another 18 fruits harvested together with the rest of the fruits used in the treatments.

### 2.2. Quality Attributes Evaluation

#### 2.2.1. Weight Loss

Weight loss was measured according to Melgar et al. [11], and batches consisting of 18 fruits per treatments were weighted on sampling day. After weighing, tomatoes were returned to their original storage conditions. Weight loss was calculated in relation to the weight of the fruit on the first day of the experiment (*n* = 18).

#### 2.2.2. Colour and Firmness

The colour of fruits was assessed using a Minolta colorimeter CR200 (Minolta Camera, Osaka, Japan) and was quantified using a CIELab colour space system, where L* represents the lightness from black 0) to white (100); a* represents green-red tonalities and b* represents yellow–blue tonalities. The L*, a*, and b* parameters were taken as the means of three determinations for each fruit along the equatorial axis (*n* = 18).

The fruit tomato firmness was evaluated using a Baxlo durometer (Baxlo Instrumentos de Medida y Precisión, S.L., Polinyá, Barcelona, Spain) This is a type of device that measures the firmness of a fruit by pressing a flat circular probe of ∅ 5 mm into its surface, measuring the force required to make an indentation. This method does not tear the skin of the fruit, and thus is a non-destructive measure of firmness. Each fruit was tested three times along the equatorial axis (*n* = 18).

#### 2.2.3. Ethylene Production

The ethylene production rate was assessed every sampling day. According to Duque et al. [2], 4 replications of 3 fruits per treatment were held in 3.5-litre sealed containers hermetically sealed with a rubber stopper for 1 h at 20 °C. Then, a sample of the gas was taken with a syringe, and the ethylene content was determined three times using a gas chromatograph (Varian 3900 GC) equipped with a flame ionisation detector (FID). The ethylene production rate was expressed in nanolitres per gram per hour (nL·g^−1^·h^−1^) (*n* = 12).

#### 2.2.4. Total Soluble Solids, Total Phenolic Content (TPC), Ascorbic Acid, Chlorophylls and Carotenoids

Twelve fruits from each treatment were mixed and mashed and homogenized. This blend was used for the analytical determination of following parameters:

Total soluble solids were determined with a temperature-compensated digital refractometer (model Atago PR-101, Atago Co. Ltd., Tokyo, Japan), and the results are expressed in Brix (*n* = 12).

Total phenolic content (TPC) was determined using the Folin–Ciocalteau reagent as reported by Singleton and Rossi [26]. Briefly, 10 g of the above blend was homogenized in 10 mL 70% aqueous methanol (70%). Then, the mixture was centrifuged (5000 rpm) for 10 min at 4 °C and the supernatant collected. Next, 100 μL of supernatant was mixed with 5 mL of Folin–Ciocalteau and 4 mL of Na_2_CO_3_ (7.5%, *w*/*v*). The mixture was incubated at 40 °C for 30 min, and its absorbance was measured at 760 nm. Gallic acid was used as a standard for calibration curve. The phenolic content was expressed as mg gallic acid per 100 g^−1^ F.W. (*n* = 12).

Ascorbic acid was determined using the 2,6-Dichloroindophenol titrimetric method [27]. One gram of the sample was mixed with 10 mL of water and allowed to settle, and then 5 mL of the supernatant was titrated by the dye solution until the colour changed. Data are expressed as mg·g^−1^ F.W. (*n* = 12).

Chlorophylls, lycopene, and *β*-carotene were determined according to the Duque et al. [2]. Then, 1 g of blended tomatoes was mixed with 15 mL of acetone:hexane 4:6 (*v:v*, after vigorous shaking, the two phases were automatically separated, then the optical density values at 663, 645, 505, and 453 nm were measured in a spectrophotometer). The total chlorophyll, lycopene and *β*-carotene contents were calculated according to the method by Nagata and Yamashita [28] and are expressed as μg·g^−1^ F.W. (*n* = 12).

### 2.3. Sensory Evaluation

Sensory evaluation was performed using an analytical–descriptive test to discriminate the sensory quality attributes between different treatments. This analysis was carried out by 10 trained participants (50% female and 50% male) aged 30–60 years. Before the experiment, the panellists were trained by evaluating highly favourable sensory attributes of tomato fruits, such as appearance, crunchiness, juiciness, sweetness and acidity. The analysis was performed both at the beginning of the experiment and on each sampling day under conditions where the panellists were separated from each other in individual booths. The panellists were instructed to rinse their mouths with distilled water, taste the tomato fruit and give their evaluation, and then rinse their mouths again before the next tasting. Each panellist evaluated a representative sample of tomatoes from each treatment and sampling time, assessing the following sensory characteristics: crunchiness, juiciness, sweetness, acidity and overall quality on a scale of 1 to 5, where 1 = extremely low, 2 = low, 3 = medium, 4 = high and 5 = extremely high. In addition, a binary scale (yes or no) was used to assess the presence of off-flavours and odours (*n* = 6).

### 2.4. Statistical Analysis

Once the data were obtained, they were checked for normality using the Kolmogorov–Smirnov test. The statistical significance of the treatments was assessed by an analysis variance (ANOVA), with storage time and treatments as sources of variation. When the F-test of the ANOVA was significant, means were compared by a post hoc LSD test. All statistical analyses were performed with a Statgraphic Centurion XVI (STATGRAPHICS. Statpoint Technologies, Inc., Warrenton, VA, USA).

## 3. Results

### 3.1. Weight Loss

**Figure 1:**
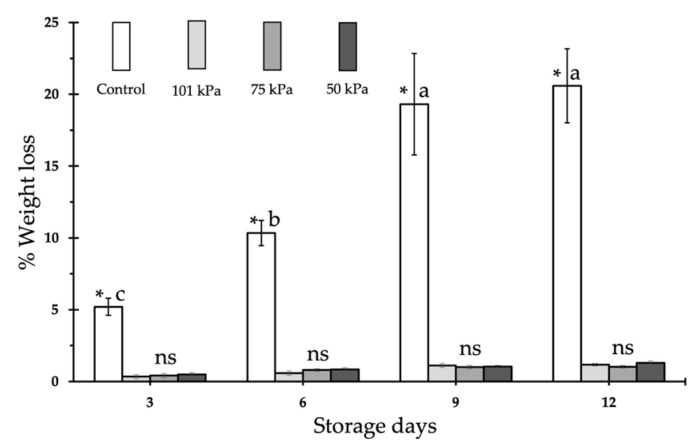
Time course of weight loss in Rojito tomato fruits under different sub-atmospheric treatments. Vertical bars show the standard error (SE) (*n* = 18). Different lowercase letters (a–c) indicate differences between days storage according to the LSD test at *p* < 0.05. Asterisk indicates significant differences within each day of storage between treatments according to the LSD test at *p* < 0.05. ns indicates statistically non-significant.

The weight loss of the tomato fruits under the different treatments is shown in Figure 1. As expected, the greatest weight loss occurred in the control fruits, as they were stored in the open air. For the rest of the treatments (101 kPa, 75 kPa and 50 kPa) the weight loss was very small, never reaching more than 2.5%, while after 3 days of storage, the control fruits had already lost a little more than 5% of their original weight, reaching a weight loss of over 20% by the end of the experiment. As expected, the control fruits showed a gradual weight loss as the storage time increased, being the weight loss statistically significant between days 3 and 6 of storage, with no significant differences in weight being found between days 9 and 12 of storage, where the fruits lost 18% and 21%, respectively. On the other hand, in the 101 kPa, 75 kPa, and 50 kPa, treatments, no significant differences were found, neither for the storage time nor between the different treatments.

### 3.2. Ethylene Production and Colour Evolution

**Figure 2:**
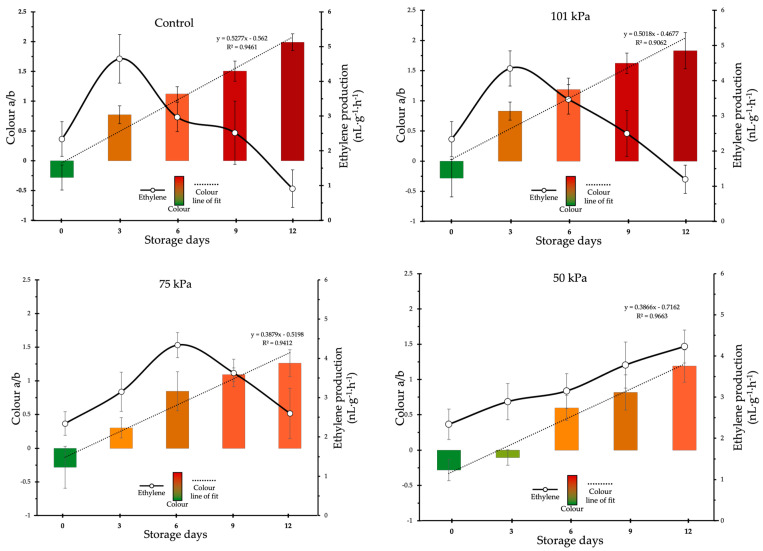
Time course of the ethylene production rate and colour (a*/b*) for Rojito tomatoes under different sub-atmospheric treatments. Vertical bars show the standard error (SE) (*n* = 12 and *n* = 18 for ethylene and a/b, respectively).

Figure 2 shows the effects of the control, 101 kPa, 75 kPa and 50 kPa treatments on the colour parameter a/b and the rate of ethylene production. The results report that a delay in ripening was found in treatments 75 kPa and 50 kPa, evidenced by a delay in the reddish colouration of the tomatoes. Similarly, a delay in the appearance of the ethylene production peak was found, and the maximum amount of ethylene produced was significantly lower in fruits subject to 75 kPa and 50 kPa, than the rest of the fruits, while the production of ethylene was similar between the control and the fruits of treatment 101 kPa.

The colour evolution of the control fruits was rapid, reaching the parameter a/b values close to 1 in the first three days of conservation and exceeding this value in 6 days. This meant that the fruits in the control treatment were already red after three days of storage. This same behaviour was exhibited by the fruits in the 101 kPa treatment; however, in treatments 75 kPa and 50 KPa, the value of the parameter a/b did not reach figures close to 1 until 9 and 12 days, respectively. Even in the 50 kPa treatment negative values were found during the first three days of storage. The slow colour change as caused by sub-atmospheric treatments can be seen by comparing the tangents of the line of fit from parameter a/b, which presented values of 0.528 (R^2^ = 0.946) and 0.502 (R^2^ = 0.906) for fruits kept in the open air and 101 kPa, respectively, while the slope of the line of fit for sub-atmospheric treatments was between 0.388 (R^2^ = 0.941) and 0.387 (R^2^ = 0.966).

### 3.3. Fruit Pigment Evolution

**Figure 3:**
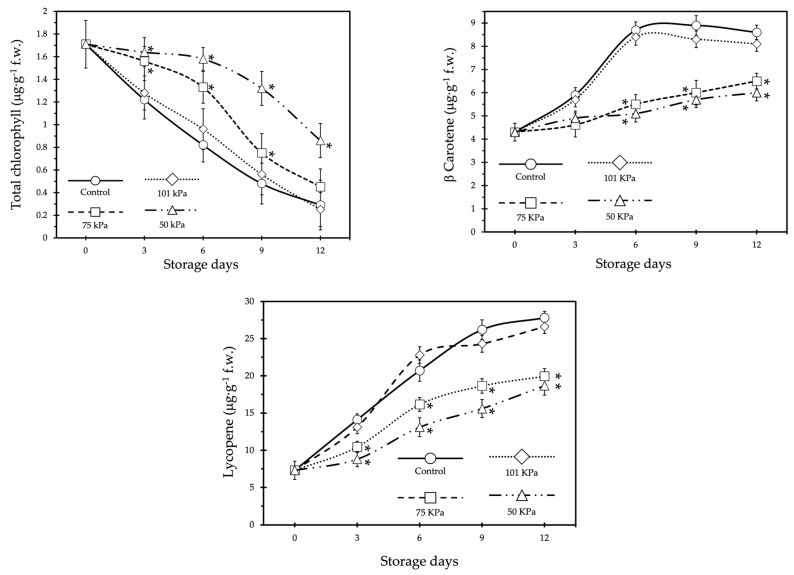
Time course of pigments content in Rojito tomatoes under different sub-atmospheric treatments. Vertical bars show the standard error (SE) (*n* = 12). Asterisk indicates significant differences within treatments according to the LSD test at *p* < 0.05. The lack of a symbol indicates no differences.

Figure 3 shows the time course of the main pigments in the studied fruits. As expected, chlorophyll content decreased, while carotenoids content increased in all treatments during postharvest time. However, a clear effect of sub-atmospheric storage was observed. Significant differences in the evolution of all pigments could be seen between sub-atmospheric treatments (75 kPa and 50 kPa) as well as open-air and 101 kPa treatments. A stronger breakdown of chlorophyll was observed in the open-air and 101 kPa treatments. From the third day of storage, significant differences in the content of all pigments could be observed between the control and 101 kPa treatments and the sub-atmospheric treatments. No differences were found between the 75 kPa and 50 kPa treatments.

### 3.4. Fruit Quality Characteristics

Table 1 shows the content of SST, firmness TPC and AAC of fruit during the postharvest period. No statistical differences were found, either between storage time or between treatments for SSC, and the fruits had a value that ranging between 7.4 and 8.2 °Brix. However, significant differences were found for firmness, total phenolic content, and ascorbic acid content. Rojito fruits, regardless of treatment applied, experienced a loss of firmness throughout the storage period. This lack of firmness was more marked in the control fruits and in those stored at 101 kPa than in the sub-atmospheric treatments. In the fruits tested under these sub-atmospheric treatments, the loss of firmness was more gradual, with differences found only at 9 and 12 days of storage with respect to the firmness of the fruits at the moment of harvest. No differences were found between 75 and 50 kPa treatments at any time during the storage period, and the fruits of these two treatments showed a significantly higher firmness than the control and 101 kPa-treated fruits from the sixth storage period onwards. Phenol content was affected by sub-atmospheric treatments, finding that phenol content was higher in control fruit and fruit stored at 101 kPa from the 6th day of storage. Ascorbic acid content decreased as storage time elapsed, but the decrease was greater in the control fruit and in fruit stored at 101 kPa from the 6th day of storage. As the storage time was longer, the ascorbic acid content decreased, however the decrease was greater in the control and 10 kPa fruits, finding significant differences between the time of harvest and the end of the experiment for these two treatments.

### 3.5. Sensory Analysis

**Figure 4:**
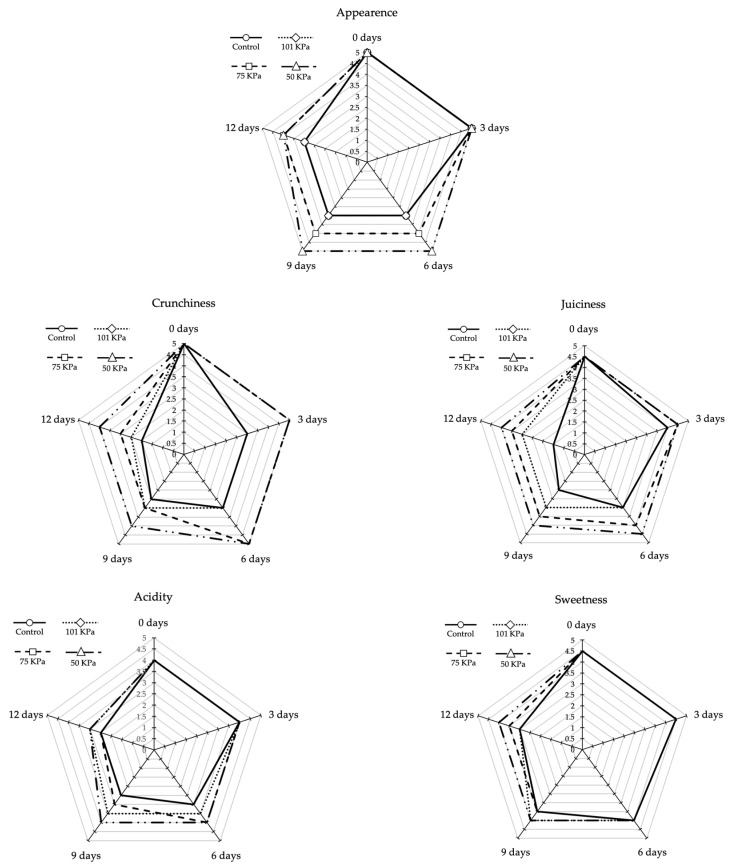
Scores for sensorial analysis throughout storage time in in Rojito tomatoes under different sub-atmospheric treatments. (*n* = 6).

Figure 4 shows the sensory analysis carried out by the judges during the storage period. A positive influence of 50 kPa storage was observed in all the analysed parameters. The fruits of this treatment had the highest values in all parameters. Thus, the appearance of these fruits was valued with the same score as at the time of harvest, during 9 of the 12 days that the experiment lasted. The control and 101 kPa treatments demonstrated the worst results, although they differed in juiciness, acidity, and crispness because in the 101 kPa treatment the panellists rated them with higher score. Note that the panellists did not find any off-flavours and odours related to fermentative processes.

**Figure 5:**
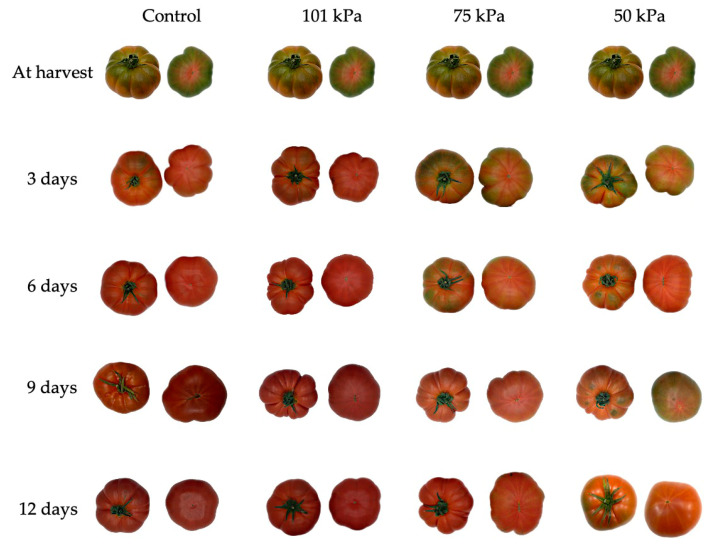
Effects of sub-atmospheric treatments on tomato fruit.

Figure 5 shows the effects of sub-atmospheric treatments. The control fruits effectively enter a state of maturity after 3 days, presenting an intense red colour after 12 days of conservation, indicating over-ripeness. A similar behaviour was observed in the 101 kPa treatment. In contrast, the sub-atmospheric treatments induced a delay in the ripening of the fruit, especially the 50 kPa treatment.

## 4. Discussion

The different cultivars of Marmande tomatoes, including Rojito, have one characteristic in common: their postharvest life is very short; therefore, they are a perishable product [29]. Moreover, they are considered gourmet products mainly intended to be consumed fresh, losing much of their commercial value when they reach the red ripe stage. Essentially, consumers most enjoy this fruit at the mature green, breaker or turning stages because this is when Marmande tomato has organoleptic characteristics of crispness and balance between sugars and acids that make it delicious. A rapid ripening implies a significant loss of commercial quality; as they ripen, the fruits become softer, take on an intense red colour and lose their juiciness. The applied sub-atmospheric treatments show that these changes can be delayed, as they are associated with a lower ethylene production and a delay in the appearance of their maximum peak.

In the sub-atmospheric and 101 kPa treatments, fruits were enclosed in a container that limited their weight loss; therefore, no differences were found between the applied treatments or between the different samples (Figure 1). As expected, when stored outdoors, the control fruits showed a weight loss that gradually increased as the storage time increased. These results are similar to those found for strawberries by Hashmi et al. [30] and those of Kou et al. [31], who did not find an increasing weight loss in tomato maintained at a very low pressure. Our results contradict those obtained for tomato by Pristijono et al. [12] who found a lower weight loss in 4 kPa than 101 kPa treatments, but we must consider that these authors applied pressures lower than one order of magnitude (i.e., around ten time less) than those applied in our experiment, which might explain the differences.

The effects of sub-atmospheric treatments on ethylene production have long been established. Vithu and Moses [14] demonstrated that there is a decrease in ethylene biosynthesis when fruits are subjected to low pressure. In addition, Kou et al. [31] found that there is a reduction in ethylene production in tomatoes when stored at low pressure. These effects have not only been observed in tomato, but also in mango and in apple [32] who found that ethylene production was much lower in fruits subjected to 50 kPa. Kou et al. [31] propose that low pressure storage causes a decrease in the respiratory rate that negatively influences ethylene biosynthesis, resulting in lower ethylene production and a delay of the climacteric peak, but it was also previously speculated that low pressure treatment removes endogenous ethylene from fruits through diffusion, limiting its physiological effects [33] Purvis and Barmore [34] demonstrate that ethylene diffusion rate dropped when the fruits were exposed at sub-atmospheric pressures, but under these conditions, the induction of chlorophyllase activity was also considerably reduced. As our fruits were at the turning stage, which usually coincides with the pre-climacteric stage [35], it is possible that the effect of lowering the respiratory rate is more influential than the removal of endogenous ethylene. The transition from chloroplast to chromoplast is responsible for the colour change during the ripening of tomato fruits. This transition is closely related to the climacteric behaviour of the fruit, and thus to the presence of ethylene. In this transition, changes take place in the chloroplast structure, as well as biochemical changes involving the degradation of chlorophyll pigments and the synthesis of carotenoids. Most of the genes involved in the chloroplast–chromoplast transition are ethylene-induced [36,37,38]; therefore, reduced ethylene synthesis induced by sub-atmospheric treatments delays chromoplast differentiation. causing the rate of colour change in tomato fruits to decrease. As the effect of sub-atmospheric conditions on colour is observed, its effect on pigment content is observed. Delaying changes in pigment content induced by sub-atmospheric pressure offers a commercial advantage for this type of tomato, as it is highly appreciated by the consumer at the turning stage. The data coincide with those found in other tomato varieties and other horticultural products. Similarly, the evolution of the pigment content in the fruit preserved in the open air is as expected for this tomato variety, and the results are in line with those described by Zhu et al. [39].

Consumers not only appreciate the colour in the quality of the Rojito tomato, but also its sweetness and taste. The sub-atmospheric treatments did not alter the balance between sweetness, acidity and flavours that is so appreciated by the consumer (Table 1). Interestingly, the 75 kPa and 50 kPa treatments delayed the softening of the fruit, resulting in a greater sensation of crispness by the panellists (Figure 4), but neither the soluble solids nor the ascorbic acid content was affected. Therefore, the organoleptic characteristics that distinguish the high quality of this cultivar were maintained for a longer period. However, the total phenol content was affected, especially in the 50 kPa treatment, since as the storage period increased, the phenol content was lower. This result may be caused by delayed ripening events, which is explained by the fact that the fruits stored at low pressure (50 kPa) had a lower respiration rate than fruits stored at atmospheric pressure, leading to a delay in the ripening process and, consequently, a decrease in phenol content. Phenolic compounds have antioxidant activity and cooperate by relieving oxidative stress induced by biochemical changes that occur during the ripening process [19,22]. Slower ripening may mean that the increase in ROS is also slower, which may explain the lower phenol content in the fruits stored under sub-atmospheric pressures.

It can be assumed that there is a real possibility that hypobaric treatment may also be helpful for reducing fruit and vegetable wastage. It is assumed that that in industrialized countries a very high percentage of post-harvest side is due to consumer and retailer waste. In this sense and household waste contributes up to 28% of total food losses [40]. It has been established that storage at sub-atmospheric pressures reduces fruit and vegetable spoilage. In our experiment, no pathogen damage was found 12 days after harvest; therefore, we believe that this method can reduce fruit wastage, especially in Marmande tomato types, which show a significant decrease in organoleptic and commercial quality once they reach the overripe stage.

## 5. Conclusions

The results of this study indicate that storing Rojito tomatoes at low pressure can be a useful strategy for prolonging their shelf life and preserving their quality. Our experiment was carried out at room temperature, and under these conditions, the data indicate that tomatoes preserved in conditions of 50 kPa maintain their organoleptic and commercial quality longer than fruits preserved in the open air. It is clear to us that implementing this technique with cold storage can increase the postharvest life of this type of fruit. Therefore, further research should be conducted in order to identify the best combinations of refrigeration and sub-atmospheric storage that would be useful for further increasing the postharvest life of fruits, without negatively affecting their organoleptic and commercial quality.

## Figures and Tables

**Table 1:** Effect of treatments on solid soluble content, firmness, total phenolic content and, ascorbic acid Content in Rojito tomato fruits.

	Harvest	3 Days	6 Days	9 Days	12 Days
Solid Soluble Content (°Brix)					
Control	8.0 ± 0.2 a A	7.6 ± 0.2 a A	7.4 ± 0.3 a A	7.6 ± 0.2 a A	7.7 ± 0.2 a A
101 kPa	8.0 ± 0.2 a A	7.7 ± 03 a A	7.6 ± 0.2 a A	7.8 ± 0.3 a A	7.9 ± 0.3 a A
75 kPa	8.0 ± 0.2 a A	7.9 ± 0.2 a A	7.8 ± 0.4 a A	7.8 ± 0.3 a A	8.1 ± 0.1 a A
50 Kpa	8.0 ± 0.2 a A	7.9 ± 0.1 a A	7.6 ± 0.3 a A	8.0 ± 0.2 a A	8.2 ± 0.3 a A
Firmness (shore hardness)					
Control	82.30 ± 1.91 a A	76.17 ± 2.18 b A	62.50 ± 3.22 c B	60.17 ± 2.40 c B	54.17 ± 3.32 d C
101 kPa	82.30 ± 1.91 a A	75.83 ± 2.17 b A	75.67 ± 4.26 b A	66.33 ± 2.84 c B	60.00 ± 3.32 c B
75 kPa	82.30 ± 1.91 a A	78.33 ± 3.52 a A	79.50 ± 2.36 a A	73.17 ± 1.19 b A	66.50 ± 3.32 c A
50 Kpa	82.30 ± 1.91 a A	79.50 ± 2.34 a A	80.83 ± 2.39 a A	75.33 ± 1.78 b A	69.17 ± 0.91 b A
Total Phenolic Content (mg·100 g^−1^ F.W.)					
Control	42.5 ± 3.8 e A	57.8 ± 2.8 d A	63.7 ± 1.9 c A	87.5 ± 2.0 a A	71.6 ± 3.1 b A
101 kPa	42.5 ± 3.8 e A	55.7 ± 1.9 d A	66.3 ± 2.6 c A	82.8 ± 3.2 a A	77.3 ± 3.4 a A
75 kPa	42.5 ± 3.8 c A	51.2 ± 2.6 b A	58.5 ± 2.4 b B	75.2 ± 3.1 a B	73.1 ± 3.6 a A
50 Kpa	42.5 ± 3.8 c A	52.8 ± 2.4 b A	55.4 ±1.9 b B	68.8 ± 3.7 a B	67.6 ± 2.1 a B
Ascorbic Acid Content (mg·100 g^−1^ F.W.)					
Control	18.5 ± 3.7 a A	17.9 ± 1.8 a A	15.9 ± 2.3 b A	15.7 ± 2.9 b A	15.2 ± 1.9 b A
101 kPa	18.5 ± 3.7 a A	18.3 ± 2.9 a A	15.6 ± 2.8 b A	16.1 ± 2.8 b A	15.1 ± 2.3 b A
75 kPa	18.5 ± 3.7 a A	18.5 ± 2.4 a A	17.5 ± 2.5 a A	17.0 ± 2.7 a A	16.7 ± 2.1 a A
50 Kpa	18.5 ± 3.7 a A	18.6 ± 2.2 a A	18.3 ± 2.1 a A	17.8 ± 1.9 a A	17.6 ± 2.2 a A

Means followed by SE (*n* = 12). Different lowercase letters (a. b) indicate significant differences within each treatment between days of storage according to the LSD test at *p* < 0.05. Different capital letters (A. B) indicate significant differences within each day of storage between treatments according to the LSD test at *p* < 0.05.

## Data Availability

The datasets generated for this study are available on request to the corresponding author.
